# The relationship between depressive and anxious symptoms and school attendance among adolescents seeking psychological services in a public general hospital in China: a cross-sectional study

**DOI:** 10.1186/s12888-023-04813-w

**Published:** 2023-06-21

**Authors:** Guoqing Zhao, Bin Wang, Hui Li, Honghong Ren, Zhian Jiao

**Affiliations:** 1 Department of Psychology, Shandong Provincial Hospital affiliated to Shandong First Medical University, Jinan, 250021 Shandong China; 2 Department of Psychology, Shandong Provincial Hospital, Shandong University, No. 324, Jingwu Weiqi Road, Huaiyin District, Jinan, 250021 Shandong China; 3 Department of Psychology, Qilu Hospital Of Shandong University Dezhou Hospital, Dezhou, 253014 Shandong China

**Keywords:** Depressive symptoms, Anxious symptoms, Adolescents, School attendance problems

## Abstract

**Background:**

School attendance problems (SAPs), whether absenteeism or dropout, are strongly associated with poor outcomes for adolescents. We examined multiple variables that influence SAPs to identify potential leverage points for improving school attendance.

**Methods:**

Self-reported SAPs and demographic information was collected from 392 adolescents in adolescents presenting to the general hospital for psychological services. PHQ-9 and GAD-7 were applied to assess the severity of depressive and anxious symptoms. We constructed logistic regression analysis and the Chi-Square Automatic Interaction Detection (CHAID) segmentation analysis via SPSS Decision Tree to identifying risk factors for the development of SAPs in adolescents.

**Results:**

SAPs were self-reported by 252 (64.3%) adolescents. The SAPs group and non-SAPs group showed a significant difference in age, PHQ9 total scores, GAD7 total scores, schools, siblings, residence, parental marital quality, general health, regular exercise, and regular diet. A post hoc comparison between the two groups showed that the frequency of SAPs was significantly higher in the moderately-severe and severe depressive groups compared with other three groups (none, mild, moderate). The frequency of SAPs in severe anxious groups was significantly different from the none-anxious group. According to the binary logistic regression analysis, the depressive severity, siblings, residence, marital quality of parents, general health, and regular diet were correlated with the SAPs among adolescents. The adjusted OR of SAPs according to moderately-severe depressive symptoms was 10.84 (95%CI: 1.967–59.742) and severe depressive symptoms was 6.659 (95%CI: 1.147–38.666). In the decision tree model, PHQ-9 severity was extracted as the first splitting variable, with regular exercise and residence as the second, and siblings as the third. The ROC curves for predicting SAPs showed a fair diagnostic accuracy of the model with AUCs of CHAID model (0.705,95%CI:0.652–0.759, *P* = 0.000) and logistic regression model (0.777,95%CI:0.729–0.824, *P* = 0.000).

**Conclusion:**

Our study provides insights into the associations between depressive symptoms and poor school attendance and identifies a number of risk factors associated with SAPs. Effective intervention by mental health practitioners, more attention by policy makers, and further research in this area are urgently needed for adolescents.

**Supplementary Information:**

The online version contains supplementary material available at 10.1186/s12888-023-04813-w.

## Background

Considering that adolescents spend a significant portion of their time at school, schools play an essential role in the physical and mental development of adolescents. Nevertheless, due to external or internal factors, some adolescents may be prone to exhibit school attendance problems (SAPs) [1]. Studies in developed countries such as Denmark, Australia, Germany, the United Kingdom, and the United States have found that the prevalence of absenteeism ranged from 5 to 25% [2, 3]. Absence from school or truancy is associated with an elevated risk of stopping one's educational career and is accompanied by an increased risk of leading to multiple social, educational, and lifelong socioeconomic disadvantages, even mental disorders [4, 5].

Individuals exhibit SAPs for reasons other than those of an unfavorable family environment (e.g., parental divorce, poverty) and school environment (e.g., bullying, poor relationship with climates), and sometimes for personal reasons, such as having a psychiatric disorder. Epidemiologic information about SAPs suggested that physical and mental health problems of students or their families were the sole or contributing cause of this behavior in more than 50% of cases [6, 7]. Older youths, youths with mental health problems, and youths whose parents had mental health problems exhibited higher levels of absence, and lower levels of non-excused absence were found among youths with highly educated fathers and youths living with both parents [2]. Mental disorders like social phobia, separation anxiety disorder, depression, or conduct disorder were associated with SAPs reported in previous studies. Adolescents who were screened positive for anxiety, depression, peer problems, and serious deficits were reported to be four to eight times more likely to miss school than their peers who screen negative [8]. Anxiety, depression, and emotional difficulties were associated with higher rates of all types of absence and the strongest association was observed for depression and unauthorized absence [9]. Meta-analyses also demonstrated small to moderate positive cross-sectional associations between depression and absenteeism as well as depression and unexcused absences/truancy [10].

Whether SAPs was a potential risk factor for psychopathology or a distinct psychosocial problem, it needed to be taken seriously enough, especially during the post Corona Virus Disease 2019(COVID-19) pandemic era and in the context of the rising prevalence of mental illness among Chinese adolescents [5, 11, 12]. The potential negative health consequences of COVID-19 pandemic far outweigh those caused by the virus itself- including severe psychological distress [13]. Adolescents who already had psychiatric disorders may be particularly vulnerable to pandemic-related distress-for example, due to fear of the virus and the major social changes (social distancing and isolation) that were initiated to minimize the spread of the virus. One study reported that long COVID was also associated with psychiatric disorders, new-onset psychiatric disorders, and suicide risk [14].

*The COVID-19 pandemic and associated restrictions have affected the normal lives of school children worldwide. In a review, the authors mentioned that children may experience impairment in daily activities and increased school absenteeism after COVID-19 *[15]*. It was also reported that student attendance during the COVID-19 pandemic was affected by a number of complex factors, including COVID-related anxiety, difficulty adjusting to new school routines, poor home-school communication and collaboration, and concerns about academic catching up *[13]*.*

Although the impact of COVID-19 on the mental health of adolescents has emerged, there was a lack of research on current status and risk factors associated with SAPs among Chinese adolescents seeking mental health services following the outbreak. Using data from 6435 Chinese middle and high school adolescents, the researchers found that the prevalence of depressive symptoms was up to 17.7% [16].

*In China, it was reported that the individuals with mental disorders preferred visiting a psychiatrist in a general hospital to visiting a physician in a psychiatric hospital (44% vs. 17%) *[17]*. Hence, there was a strong necessary to study the mental health status of adolescents who seeking psychological services in public general hospitals, considering the help-seeking preferences of individuals and the large population size in China.*

Up to now, few studies have directly examined the effects of COVID-19 on SAPs, academic performance, or child mental health and functioning [11]. In China, with the increasing pressure of social competition and the impact of the COVID-19 pandemic, SAPs have become more common among teenagers. In this study, we attempted to explore the factors influencing SAPs of adolescent students seeking mental health services at a general hospital and the relationship between depressive and anxious symptoms and SAPs. We hypothesized that SAPs were associated with depressive and anxious symptoms, even after adjusting for some variables (e.g., sex, age, and schools).

## Methods

### Participants

Participants aged 12 to 17 years visiting to the psychology department of Shandong Provincial Hospital for psychological services were recruited from June 2021 to May 2022. The study was reviewed and approved by the local ethics committee at Shandong Provincial Hospital affiliated to Shandong First Medical University (SWYX:NO2021-312). All protocols related to human experiments were conducted following the Declaration of Helsinki. All participants and their guardians received a detailed explanation of the study and written informed consent was obtained from all individuals before their inclusion. We made sure all of them knew the potential risks and benefits of our research before their participation. The inclusion criteria for participation were as follow: (a) adolescents aged 12 to 17 years; (b) presenting to the psychology department of the hospital for psychological services; (c) no physical illness in the last three months; (d) no online teaching in the last three months due to the impact of the COVID-19 wave; (d) obtained informed consent and signatures for the participation of this study from adolescents and their guardians.

### Measures

Demographic data (age, gender, schools (junior high school or senior high school), siblings (single or non-single), residence (rural or urban), marital quality of parents (bad, fair or good), general health (bad, fair, or good), regular exercise (no or yes), regular diet (no or yes), history of the mental illness (no or yes), history of the somatic disease (no or yes), dietary habit (vegetarianism, balance, or meat-eater) was collected by having adolescents fill out a collection sheet.

Patient Health Questionnaire-9(PHQ-9) and Generalized Anxiety Disorde-7(GAD-7) were used to assess the severity of depressive and anxious symptoms in adolescents, respectively. The PHQ-9 is a 9-item scale with scores that range from 0 to 27 and the GAD-7 is a 7-item scale with scores that range from 0 to 21. The answers to each question on both scales include four identical options: “not at all (0), several days (1), more than half the days (2), or nearly every day (3)”. The total PHQ-9 score and the total GAD-7 score were used to assess the overall severity of depression with higher scores indicating greater symptom frequency. PHQ-9 scores of 5, 10, 15, and 20 represented mild, moderate, moderately-severe, and severe depression, respectively [18]. GAD-7 scores of 5, 10, and 15 represented mild, moderate, and severe anxiety, respectively [19]. The 32-item Hypomania Checklist (HCL-32) and the Mood Disorder Questionnaire (MDQ) was used for excluding the possibility of bipolar disorder in adolescents.

*Studies in the Chinese population have shown that the Chinese versions of the PHQ-9 and the GAD-7 had good reliability and validity* [20, 21].

*There were four types of SAPs: school refusal, truancy,* school withdrawal, and school exclusion [22]. Various labels have been used to describe different presentations of SAPs, such as “truancy” (school absences due to a lack of motivation, often accompanied by externalizing symptoms), “school refusal” (school absences because of internalizing symptoms such as anxiety, psychosomatic complaints, or depression), and “problematic school absenteeism” (student is absent to a “problematic” degree, e.g., 25% of school time in 2 weeks or 10 days in 15 weeks, for illegitimate reasons other than parental withdrawal from school). In consideration of the heterogeneity of SAPs regarding etiology and presentation, the inconsistency of the criteria used in many studies [2, 9, 10], as well as the local situation in China, in this study, SAPs was used as a status description for students who failed to attend school on time (excluding particularly serious physical illness and online teaching because of the epidemic), whether they were absent for legitimate reasons or dropped out. Students who have to stay in school for whatever reason were considered to have no SAPs. SAPs of adolescents in the last three months was collected by asking adolescents to answer the questions: “Which of the following does your school attendance for the last three months match? Attended school on time (never took time off); Occasionally took time off (Whether Authorized or Unauthorized); Frequently took time off (Whether Authorized or Unauthorized); Suspended from school; Abandoned school or school refusal”. Students who chose to attend school on time were considered to be in the non-SAPs group and the rest were classified as SAPs group.

### Procedure

A cross-sectional observational design was used to analyze data collected in Shandong Provincial Hospital, Jinan, Shandong, China. Advertisements for recruitment of participants were posted in the waiting room of the psychology department of the hospital. Subjects and their guardians who agreed to participate in this study signed a paper version of the informed consent form prior to enrollment. Participants were directed to complete a battery of web-based questionnaires including school attendance, demographic information, and mental health measures prior to the consultation. The initially recruited participants comprised 592 individuals. The exclusion criteria were as follows: participants < 12 years old (*n* = 4); participants ≥ 18 years old (*n* = 43); HCL-32 ≥ 13 (*n* = 113); MDQ ≥ 6 (*n* = 25); ethnic minorities (*n* = 6); primary school students (*n* = 6); missing variables or values (*n* = 3). Ultimately, 394 participants were included in the study.

### Data analysis

In our study, SPSS 22 for Windows was used for statistical computations. Demographic data and mental health measures were analyzed using chi-squared or one way analysis of variance (one-way ANOVA) as appropriate. Binary logistic regression analysis was performed to estimate odds ratios (ORs) and 95% confidence intervals (CIs) by adjusting various covariates. We constructed the Chi-Square Automatic Interaction Detection (CHAID) segmentation analysis via SPSS Decision Tree to identifying risk factors for the development of SAPs in adolescents. The cut-off value of root node or daughter node was automatically selected according to the most significant P-value. Diagnostic categorical variable was 0 = non-SAP, while the positive actual state was 1 = SAPs. The tree specified at least 100 cases per parent node and 50 cases per daughter node. Nodes were split using the Pearson chi-square test when *P* < 0 0.05. After the initial tree was constructed, it needed to be ensured that the leaf nodes of the tree were minimized to classify effectively on the premise of good precision. Finally, parameters from the regression models were used to conduct the Decision Tree.

## Results

Out of a total of 392 adolescents, non-SAPs group had 140 (35.7%) adolescents attended school on time within the last 3 months and SAPs group had 252 (64.3%) adolescents. Within the SAPs group, the number of adolescents who occasionally took time off, frequently took time off, suspended from school, and abandoned school or school refusal was 99 (25.3%), 117 (29.8%), 22 (5.6%), and 14 (3.6%), respectively. As shown in Table 1, the two groups showed significant differences in age, PHQ-9 total scores, GAD-7 total scores, schools, siblings, residence, marital quality of parents, general health, regular exercise, and regular diet. No significant difference was found in gender, psychiatric history, somatic medical history, and dietary habits between the SAPs group and the non-SAPs group.

**Table 1:** Characteristics of 392 adolescents

	Total (*N* = 392)		SAPs (*N* = 252,64.3%)		non-SAPs (*N* = 140,35.7%)		*F/χ*^*2*^	*p-*value
	N/mean	%/sd	N/mean	%/sd	N/mean	%/sd		
Age	15.10	1.474	15.21	1.448	14.90	1.504	4.122	**0.043**^a^*
Gender								
Female	246	62.8	161	65.40	85	34.60	0.388	0.533^b^
Male	146	37.2	91	62.30	55	37.70		
GAD-7	12.78	5.197	13.40	4.953	11.66	5.452	10.335	**0.001**^a^*
PHQ-9	16.70	6.399	18.02	5.702	14.32	6.897	32.566	**0.000**^a^*
School								
Junior high school	156	39.8	88	56.4	68	43.6	7.000	**0.008**^b^*
Senior high school	236	60.2	164	69.5	72	30.5		
Siblings								
Single	117	29.8	64	54.7	53	45.3	6.674	**0.010**^b^*
Not single	275	70.2	188	68.4	87	31.6		
Residence								
Rural	108	27.6	84	77.80	24	22.20	11.819	**0.001**^b^*
Urban	284	72.4	168	59.20	116	40.80		
Marital quality of parents								
Bad	58	14.8	34	58.6	24	41.4	7.104	**0.029**^b^*
Fair	155	39.5	112	72.3	43	27.7		
Good	179	45.7	106	59.2	73	40.8		
General health								
Bad	56	14.3	48	85.70	8	14.30	27.310	**0.000**^b^*
Fair	255	65.1	169	66.30	86	33.70		
Good	81	20.7	35	43.20	46	56.80		
Regular exercise								
No	259	66.1	185	71.40	74	28.60	16.964	**0.000**^b^*
Yes	133	33.9	67	50.40	66	49.60		
Regular diet								
No	249	63.5	180	72.30	69	27.70	19.043	**0.000**^b^*
Yes	143	36.5	72	50.30	71	49.70		
History of mental illness								
No	360	91.8	227	63.10	133	36.90	2.907	0.088^b^
Yes	32	8.2	25	78.10	7	21.90		
History of somatic disease								
No	354	90.3	224	63.30	130	36.70	1.619	0.203^b^
Yes	38	9.7	28	73.70	10	26.30		
Dietary habit								
Vegetarianism	66	16.8	45	68.20	21	31.80	1.192	0.551^b^
Balance	255	65.1	159	62.40	96	37.60		
Meat-eater	71	18.1	48	67.60	23	32.40		

SAPs school attendance problems; PHQ-9 Patient Health Questionnaire-9; GAD-7 Generalized Anxiety Disorde-7

^a^ One-way analysis of variance (ANOVA) p-value for the difference in age, PHQ-9, GAD-7 between the two groups

^b^ Chi-square test p-value for the difference in gender, school, siblings, residence, marital quality of parents, general health, regular exercise, regular diet, history of mental illness, history of somatic disease, dietary habit between the two groups

Bold values indicate statistical significance at *p* < 0.05 level

^*^p < 0.05

**Fig. 1:**
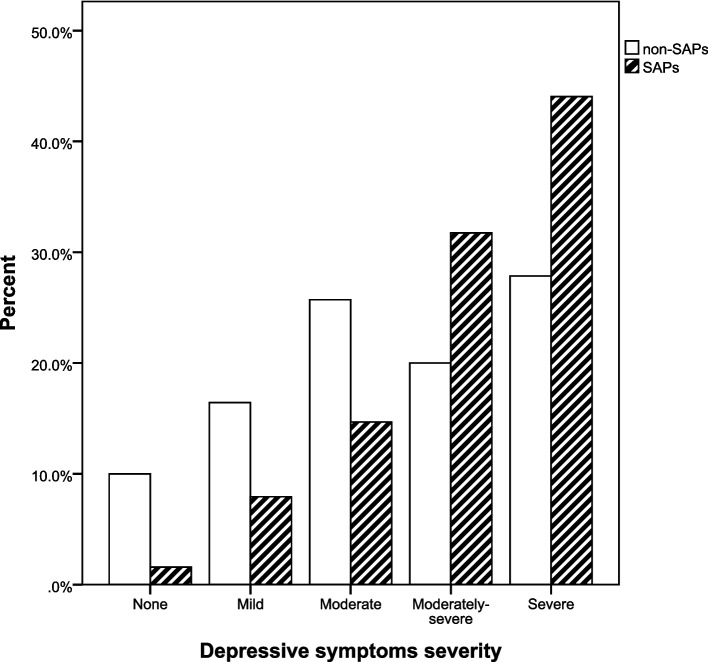
Percentage of SAPs and non-SAPs with different depressive severity. SAPs school attendance problems

**Fig. 2:**
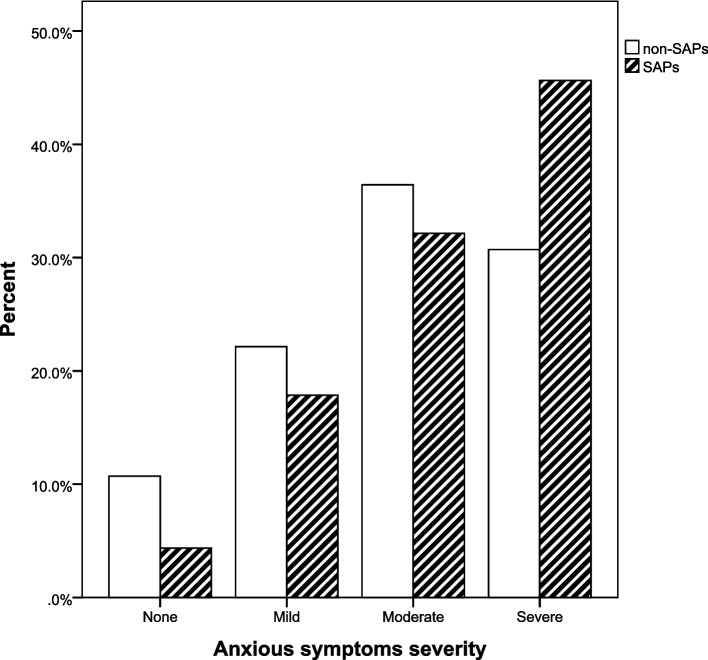
Percentage of SAPs and non-SAPs with different anxious severity. SAPs school attendance problems

Regarding mental scale scores, there were significant differences between the two groups. The distribution of SAPs among adolescents with different depressive severity (χ^2^ = 36.342, *P* = 0.000) and anxious severity (χ^2^ = 11.785, *P* = 0.000) were shown in Figs. 1 and 2. A post hoc comparison between the two groups showed that the frequency of SAPs was significantly higher in moderately-severe depressive group and severe depressive group than in the other three groups (none, mild, moderate); there was no significant difference in the frequency of SAPs between moderately-severe and severe depressive group (see Additional files 1). The frequency of SAPs in severe anxious group was significantly higher than that of the none-anxious group. There was no significant difference in the frequency of SAPs between the rest of the two comparisons (see Additional files 2).

We performed a binary logistic regression model to analysis the risk factors of SAPs entered the variables such as age, school, gender, siblings, residence, marital quality of parents, general health, regular exercise, regular diet, depressive severity, and anxious severity. According to the analysis, the depressive severity, siblings, residence, marital quality of parents, general health, and regular diet were correlated with the SAPs among adolescents, but no correlation was found between anxious severity and the SAPs. The crude ORs and adjusted ORs were shown in Table 2. The crude and adjusted ORs of SAPs according to moderately-severe depressive symptoms was 10.000(95%CI: 3.037–32.924) and 10.84 (95%CI: 1.967–59.742). And according to severe depressive symptoms, the crude and adjusted ORs was 9.962 (95%CI: 3.093–32.082) and 6.659 (95%CI: 1.147–38.666).

**Table 2:** Crude and adjusted ORs of binary logistic regression analysis to predict SAPs

	Crude			Adjusted		
	OR	95%CI(Lower)	95%CI(Upper)	OR	95%CI(Lower)	95%CI(Upper)
Age	1.155*	**1.004**	**1.329**	1.005	0.773	1.306
Gender						
Female	1(reference)			1(reference)		
Male	0.874	0.571	1.337	1.066	0.637	1.785
GAD-7						
None	1(reference)			1(reference)		
Mild	1.979	0.803	4.881	0.916	0.241	3.486
Moderate	2.166	0.923	5.084	0.532	0.132	2.139
Severe	**3.647**	**1.554**	**8.560**	0.747	0.179	3.112
PHQ-9						
None	1(reference)			1(reference)		
Mild	3.043	0.861	10.756	2.930	0.582	14.747
Moderate	3.597*	**1.081**	**11.970**	3.082	0.569	16.687
Moderately-severe	10.000*	**3.037**	**32.924**	10.84*	**1.967**	**59.742**
Severe	9.962*	**3.093**	**32.082**	6.659*	**1.147**	**38.666**
School						
Junior high school	1(reference)			1(reference)		
Senior high school	1.760*	**1.156**	**2.68**	1.947	0.886	4.275
Siblings						
Single	1(reference)			1(reference)		
Not single	1.790*	**1.148**	**2.79**	1.820*	**1.07**	**3.096**
Residence						
Rural	1(reference)			1(reference)		
Urban	0.414*	**0.248**	**0.69**	0.420*	**0.233**	**0.757**
Marital quality of parents						
Bad or very bad	0.544	0.290	1.021	0.473*	**0.235**	**0.953**
Fair	1(reference)			1(reference)		
Very good or good	0.557*	**0.352**	**0.884**	0.799	0.467	1.368
General health						
Bad or very bad	3.053*	**1.383**	**6.742**	2.681*	**1.149**	**6.256**
Fair	1(reference)			1(reference)		
Very good or good	0.387	0.232	0.645	0.772	0.414	1.440
Regular exercise						
No	1(reference)			1(reference)		
Yes	0.406	0.263	0.627	0.678	0.406	1.132
Regular diet						
No	1(reference)			1(reference)		
Yes	0.389*	**0.253**	**0.597**	0.519*	**0.306**	**0.881**

The table shows the crude and adjusted ORs of binary logistic regression analysis to predict SAPs

Bold values indicate statistical significance at *p* < 0.05 level

SAPs school attendance problems; PHQ-9 Patient Health Questionnaire-9; GAD-7 Generalized Anxiety Disorde-7; OR odds ratio; CI confidence interval

^*^p < 0.05

**Fig. 3:**
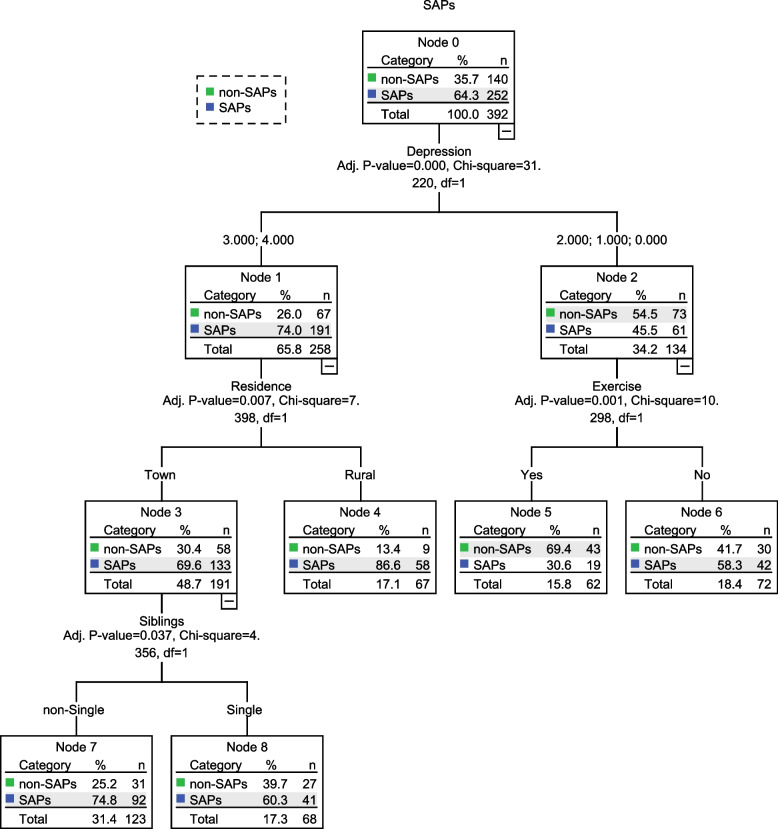
The decision tree model of predictive SAPs in adolescents. Depressive severity was extracted as the first splitting variable, with regular exercise and residence as the second, and siblings as the third. SAPs school attendance problems

In the decision tree model, depressive severity was extracted as the first splitting variable, with regular exercise and residence as the second, and siblings as the third, Fig. 3. At the first line, adolescents with moderately-severe and severe depression had a higher probability to develop into SAPs (74.0% vs. 45.5%). At the second line, among the subgroup with no, mild, and moderate depression, the adolescents with non-regular exercise yielded a higher probability of SAPs (58.3% vs. 30.6%). Among the subgroup with moderately-severe and severe depression, the adolescents from rural area yielded a higher probability of SAPs (86.6% vs. 69.6%). At the third line, adolescents with siblings who live in urbans were more likely to have SAPs (74.8% vs. 60.3%).

**Fig. 4:**
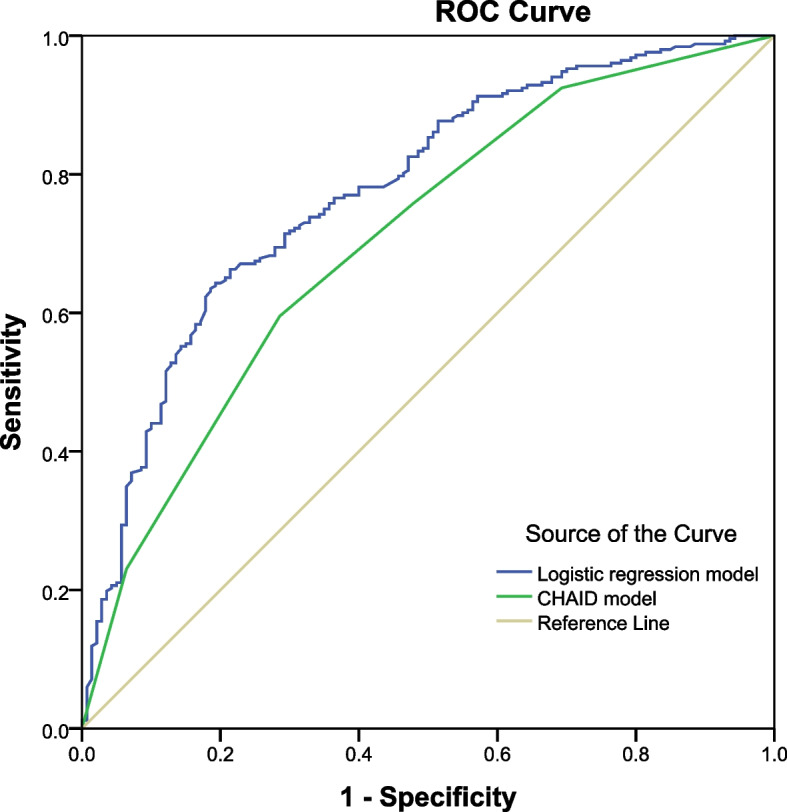
The ROC curves of the logistic regression model and the CHAID model for predicting accuracy of SAPs. SAPs school attendance problems, ROC receiver operating characteristic, CHAID Chi-Square Automatic Interaction Detection

The receiver operating characteristic (ROC) curves for predicting SAPs showed a fair diagnostic accuracy of the model with AUCs of CHAID model (0.705,95%CI:0.652–0.759, *P* = 0.000) and logistic regression model (0.777,95%CI:0.729–0.824, *P* = 0.000), which was shown in Fig. 4. It can be noted that the classification results of both models were practically meaningful and the models had some accuracy in classification.

## Discussion

SAPs have become manifest in many ways and many counties in the world. School absenteeism represented a potential risk factor for psychopathology that was a widespread and potentially preventable problem [5]. However, there is little research on school attendance among Chinese adolescents, especially in the post-COVID-19 epidemic era. This study found that 64.3% adolescents seeking psychological services at a general hospital had SAPs. This result was comparable to the previous results among treatment-seeking adolescents, but higher than that among the general student population [9, 23]. Risk factors identified through logistic regression analysis include the depressive severity, residence, siblings, marital quality of parents, general health, and regular diet. The risk factors identified in the decision tree model were depressive severity, residence, regular exercise, and siblings.

A negative relationship between depressive symptoms and years of schooling was found in previous study and it was estimated that a standard deviation increase in depressive symptoms is associated with a 25–30% increase in the likelihood of dropping out [24]. A previous study has revealed that youths experiencing both mild (adjusted OR = 1.57; 95% CI = 1.22–2.01) and severe (adjusted OR = 1.80; 95% CI = 1.04–3.13) depression (referent: no depression) increase the risk of SAPs [25]. In this study, adolescents with moderately-severe and severe depressive symptoms were more likely to have SAPs in the last three months, but no significant correlation was found in mild to moderate depression. This finding was in line with previous research showing the strong association between depression and poor attendance and also suggested that different levels of depression may have different effects on SAPs [9].

In the univariate analysis, it was found that the total GAD-7 score was significantly higher in the SAPs group than in the non-SAPs group and that the crude OR for severe anxiety leading to SAPs was 3.647(95%CI:1.554–8.560). However, no significant association was found between SAPs and anxious severity in the model after correcting for confounders in this study. Similar to the results of this study, there was no evidence that anxiety score measured using the Spence Children’s Anxiety Scale (SCAS) or Hospital Anxiety and Depression Scale (HADS) was associated with school attendance among children aged under 18 years [26]. In contrast to these results, a study had reported that anxiety was associated with higher rates of all types of absence [9]. Taken together, these results suggest that the relationship between anxious severity and SAPs needs to be further investigated. There may be other factors influencing this relationship, for example, it was found that differences in how adolescents view the various stress dimensions and mediated pathways associated with anxiety and depressive symptoms and transient anxiety partly accounted for the indirect effects of eight stress dimensions on depressive symptoms [27].

In the present study, adolescents living in rural areas were more likely to develop SAPs, especially in the moderately-severe depressive group and severe depressive group. One reason may stem from China's long-standing problem of urban–rural disparity, even in the more developed provinces. A previous study has reported that rural adolescents appeared to be more vulnerable to symptoms of anxiety and depression compared with urban adolescents in two areas of Zhejiang Province, China [28]. More than half of the rural participants aged 14–16 in mainland China reported that they felt they were not likely to attain the level of education to which they aspired, and whose aspirations exceed expectations were more likely to report lower self-esteem, higher depression, lower academic self-perception, and poorer self-regulation than those without a discrepancy [29]. Another reason may be that with the rapid development and urbanization of China in recent years, large numbers of laborers migrated from rural areas to large cities, which has led to the emergence of many left-behind youth in rural areas. In China, alienation towards parents was high in left-behind children, and 21.01% of them reported depression. Alienation towards parents predicted current depression of children directly and later depression indirectly [30]. The influence of rural residential background on adolescents’ mental health can extend even into early adulthood and further. Rural background was positively associated with depression, which was in turn associated with suicidal ideation in Chinese college students [31].

In our study, adolescents with siblings were more likely to present with SAPs, and this was more noticeable in adolescents living in urban areas. In a recent study, it was found that adolescents from one-child families were more likely to remit from depressive symptoms over time than adolescents with siblings, and having siblings significantly predicted persistent/remitted depressive symptoms in Chinese high school students [32]. Sibling depressive symptoms may be a risk factor for adolescent depressive symptoms [33]. In single child families, greater parental resources may lead to better parental guidance and individual care for single children. Such social support may help depressed children make better psychological and behavioral adjustments, which provides greater opportunity to remit from depression [34]. Moreover, this may be related to the changes in family structure due to China's reproductive planning policy adjustments. The one-child policy has been implemented in China since 1979. In October 2015, China fully liberalized its two-child policy, which was especially meaningful for urban families with one child. This has also, to some extent, led to changes in child-rearing practices among parents who formerly lived in one-child families. The influence of family environment including parenting style and number of siblings on adolescent personality traits has been confirmed in a study [35].

Whilst many risk factors such as marital quality of parents, general health, and regular diet were identified by logistic regression analysis, similar results were not verified in the decision tree model in our study sample. In the decision tree model, among the subgroup with none, mild, and moderate depression, the adolescents with non-regular exercise were more likely to present SAPs compared with those exercise regularly, however, regular exercise did not enter the logistics regression equation. One potential reason may be that although the two models were widely used in different fields, each has its own strengths and weaknesses [36, 37]. Although logistic regression reflected the dependence of SAPs on their respective variables, it did not provide a targeted analysis and visualization of the importance of each influencing factor on SAPs. The decision tree was not affected by the covariance between variables, and the variables were independent of each other in the process of extracting independent variables, and the potential interactions between influencing factors were well reflected. Another possibility was that the sample was relatively mild, and there may have been too few adolescents with SAPs and non-SAPs for the models to identify the criterion as a classifier or a risk factor.

*In China, adolescents’ mental health has become a very important public health concern. Seeking professional help from psychologists is an important way for adolescents to seek help for their psychological problems, and increasing knowledge of SAPs from psychologists will help this problem be better solved. In our present study, we found a number of factors associated with adolescent SAPs, including depressive severity, siblings, residence, marital quality of parents, general health, and regular diet. What is particular in our study is that, with the help of decision tree analysis, we found depression severity as the first splitting variable, with regular exercise and residence as the second, and siblings as the third. These findings suggested differences in the extent to which various influences affected SAPs in adolescents, with depressive symptoms playing the most important role in the present study. Our study provides new insights for psychologists to understand the potential factors influencing SAPs and suggests to psychologists the importance of dealing with depressive symptoms in adolescents with SAPs.*

### Limitations

Several limitations should be noted in our study. First, data on adolescent characteristics, including SAPs, GAD-7, and PHQ-9, were self-reported and had self-report bias. It was possible that symptoms elicited by physicians during consultations and observed by parents (or teachers) were not fully consistent with the adolescents’ self-reported symptoms. It was important to emphasize that our model represented only a statistical relationship between the presence of SAPs and depressive severity based on adolescents’ self-report. Independent collection of physician-elicited symptoms and parents (or teachers) reported SAPs and characteristics would help to validate our findings.

Secondly, although we cautiously used both HCL-32 and MDQ to help us screen for the possible presence of bipolar disorder, there was a risk of underdiagnosis. After all, in previous studies it was found that a relatively large proportion of adolescents with depression had bipolar disorder [38]. In addition, the presence of other psychotic disorders in adolescent students based on self-report methods has not been effectively excluded.

Third, this was a cross-sectional study and our results were based on 394 adolescent students seeking psychological services at a general hospital and may not be representative of other environments in which SAPs occurred. Differences in adolescents' family economic status, parental educational background, cities of living, and transportation can affect both behavior of medical visits and types of hospital visited. The decision tree approach used in our findings provided relatively important classifiers and decidedly valuable information for determining different SAPs; however, further studies in other environments were needed to deepen further knowledge of SAPs. In addition, there is a need to further expand the sample size in future studies.

## Conclusions

In conclusion, we found that nearly two-thirds of adolescent students seeking psychological services had SAPs within the last 3 months. Depressive symptoms, especially moderately-severe and severe depressive symptoms, but not anxious symptoms, were strongly associated with the emergence of SAPs in adolescents. Both models have demonstrated that adolescents with siblings and living in rural areas were more vulnerable to SAPs. We found that decision tree analysis was an informative method to explore the factors associated with SAPs in adolescents. Our findings suggested new insights regarding psychologists' knowledge of the potential factors influencing SAPs and awareness of the importance of dealing with depressive symptoms. Finally, it would seem that interventions that account for the complexity of SAPs may be more effective for adolescents together with moderately-severe or severe depressive symptoms. Future studies of sociodemographic characteristics and depressive/anxious symptoms of adolescents with SAPs are needed to validate our findings.

## Supplementary Information

**Additional file 1.** Post hoc comparisons of SAPs and non-SAPs between different depressive groups.

**Additional file 2.** Post hoc comparisons of SAPs and non-SAPs between different anxious groups.

## Data Availability

The data that support the findings of this study are available from the corresponding author, Zhian Jiao, upon reasonable request.

## Abbreviations

SAPs: School attendance problems
COVID-19: Corona virus disease 2019
PHQ-9: Patient health questionnaire-9
GAD-7: Generalized anxiety disorde-7
HCL-32: 32-Item hypomania checklist
MDQ: Mood disorder questionnaire
one-way ANOVA: One way analysis of variance
ORs: Odds ratios
CIs: Confidence intervals
CHAID: Chi-Square automatic interaction detection
ROC: Receiver operating characteristic
SCAS: Spence children’s anxiety scale
HADS: Hospital anxiety and depression scale
