# Supplementary material for: The relationship between depressive and anxious symptoms and school attendance among adolescents seeking psychological services in a public general hospital in China: a cross-sectional study
Source: BMC Psychiatry. 2023 Jun 21;23:456. doi: 10.1186/s12888-023-04813-w (PMC10286379; doi:10.1186/s12888-023-04813-w)
Supplement: Supplementary file 1 — Additional file 1. Post hoc comparisons of SAPs and non-SAPs between different depressive groups. [file 12888_2023_4813_MOESM1_ESM.docx]

| Additional File 1. Post hoc comparisons of SAPs and non-SAPs between different depressive groups | | | | | |  |
| --- | --- | --- | --- | --- | --- | --- |
|  |  | PHQ-9 severity | | | | |
|  |  | none | mild | moderate |  | severe |
| non-SAPs | N | 14^a^ | 23^a^ | 36^a^ | 28^b^ | 39^b^ |
|  | % within non-SAPs | 10.00% | 16.40% | 25.70% | 20.00% | 27.90% |
|  | % within GAD7-severity | 77.80% | 53.50% | 49.30% | 25.90% | 26.00% |
| SAPs | N | 4^a^ | 20^a^ | 37^a^ | 80^b^ | 111^b^ |
|  | % within SAPs | 1.60% | 7.90% | 14.70% | 31.70% | 44.00% |
|  | % within GAD-7 severity | 22.20% | 46.50% | 50.70% | 74.10% | 74.00% |
| Notes:  The data show Post hoc comparison of SAPs and non-SAPs between different depressive groups  Each subscript letter denotes a subset of PHQ-9 severity categories whose column proportions do not differ significantly from each other at the 0.05 level.  Abbreviations:  SAPs: school attendance problems; PHQ-9: Patient Health Questionnaire-9. | | | | | | |
