# Supplementary material for: The relationship between depressive and anxious symptoms and school attendance among adolescents seeking psychological services in a public general hospital in China: a cross-sectional study
Source: BMC Psychiatry. 2023 Jun 21;23:456. doi: 10.1186/s12888-023-04813-w (PMC10286379; doi:10.1186/s12888-023-04813-w)
Supplement: Supplementary file 2 — Additional file 2. Post hoc comparisons of SAPs and non-SAPs between different anxious groups. [file 12888_2023_4813_MOESM2_ESM.docx]

| Additional File 2. Post hoc comparisons of SAPs and non-SAPs between different anxious groups | | | | | |
| --- | --- | --- | --- | --- | --- |
|  |  | GAD-7 severity | | | |
|  |  | none | mild | moderate | severe |
| non-SAPs | N | 15^a^ | 31^a, b^ | 51^a, b^ | 43^b^ |
|  | % within non-SAPs | 10.70% | 22.10% | 36.40% | 30.70% |
|  | % within GAD7-severity | 57.70% | 40.80% | 38.60% | 27.20% |
| SAPs | N | 11^a^ | 45^a, b^ | 81^a, b^ | 115^b^ |
|  | % within SAPs | 4.40% | 17.90% | 32.10% | 45.60% |
|  | % within GAD-7 severity | 42.30% | 59.20% | 61.40% | 72.80% |
| Notes:  The data show Post hoc comparison of SAPs and non-SAPs between different anxious groups  Each subscript letter denotes a subset of GAD-7 severity categories whose column proportions do not differ significantly from each other at the 0.05 level.  Abbreviations:  SAPs: school attendance problems; GAD-7: Generalized Anxiety Disorde-7. | | | | | |
